# Pollen-Structured Gold Nanoclusters for X-ray Induced Photodynamic Therapy

**DOI:** 10.3390/ma11071170

**Published:** 2018-07-09

**Authors:** Lih Shin Tew, Meng-Ting Cai, Leu-Wei Lo, Yit Lung Khung, Nai-Tzu Chen

**Affiliations:** 1 Regenerative Medicine Cluster, Advanced Medical and Dental Institute (AMDI), Universiti Sains Malaysia, Kepala Batas 13200, Malaysia; tls16_ipg005@student.usm.my; 2 Institute of New Drug Development, China Medical University, Taichung 40402, Taiwan; 3 Department of Biological Science and Technology, China Medical University, Taichung 40402, Taiwan; qqq22605@gmail.com; 4 Institute of Biomedical Engineering and Nanomedicine, National Health Research Institutes, Zhunan 35053, Taiwan; lwlo@nhri.org.tw

**Keywords:** photodynamic therapy, pollen-structured gold clusters, reactive oxygen species, mesoporous silica

## Abstract

Photodynamic therapy (PDT) is a cancer treatment that employs the production of cytotoxic reactive oxygen species (ROS), subsequently triggering tumor apoptosis and tumor size reduction. However, this approach suffers from insufficient light penetration depth. In order to mitigate this issue, pollen-structured gold clusters (PSGCs) were designed for mediating X-ray-induced PDT for radiotherapy enhancement. The structure of PSGCs provides a large surface area that is able to generate ROS upon X-ray irradiation. The synthesized PSGCs were exposed to different X-ray doses and the generated ROS was then quantified by dihydroethidium (DHE) assay. Furthermore, at the cellular level, the PDT efficacy of PSGCs was evaluated via immunofluorescence staining with γ-H2AX and comet assay. The results demonstrated that PSGCs possess a significantly high ROS-generating capacity and a remarkable PDT efficacy in the treatment of breast cancer cells, thus showing potential clinical uses in deep-tissue cancer treatment.

## 1. Introduction

Following the rapid developments in near-infrared (NIR) laser irradiation [1,2], cold atmospheric plasma [3,4], and proton therapy [5,6], the field of non-invasive therapeutics has been steadily positioning itself as a serious alternative to conventional cancer therapies in recent years. At times, these advances have often been achieved in tandem with nanotechnology-based innovations, and of these many emerging synergistic technologies require attention to be paid to the subject of photodynamic therapy (PDT) [7,8]. PDT, also often referred as photoradiation therapy or photochemotherapy, is a new and less invasive localized cancer treatment employing three essential components: a light source, photosensitizers (PS), and oxygen molecules [9]. Upon light source irradiation, the activated PS generate cytotoxic reactive oxygen species (ROS), such as hydrogen peroxide (H_2_O_2_), singlet oxygen (^1^O_2_), hydroxyl radicals (•OH), and superoxide radicals (O_2_^−^), via either electron or energy transfer to surrounding oxygen molecules. These highly reactive species direct cancer cell apoptosis or induce the damage of tumor microvessels, leading to eventual tissue ischemia. This therapy strategy has been utilized in clinical treatments for different type of cancers, including non-small cell lung cancer, esophagus cancer, bladder cancer, and head and neck cancer [10,11].

However, one major shortcoming of PDT is the shallow tissue penetration of light, which greatly limits the permeability into tumors that are situated deep underneath the skin. Although there have been many strides made with near-infrared (NIR) lasers or two-photon lasers at the infrared wavelength to improve the penetration profile [12,13], these light sources are still deemed to be too shallow for practicality purposes (~1 cm). This issue can be partially diminished by utilizing advanced light-delivering technologies such as implanted optical fibers that allow for the illumination of internal cavities such as the esophagus, lung, or bladder [14,15]. Nonetheless, it is still considered challenging to use conventional PDT treatment on tumors of large volumes or multiple loci [16]. Such concerns have provided great impetus to use X-ray as the light source due to its deeper tissue penetration (8–14 cm) as compared to two-photon and NIR lasers [17]. An X-ray-induced PDT could offer a great advantage in treating tumors seated deep under the skin or within internal organs. So far, the use of gold-based nanoparticles as PS for mediating PDT has remained a well-studied proposition due to its bio-inertness and highly robust chemistry [18,19,20].

There are many useful attributes of the properties of gold nanoparticles in their service as PS; however, most of these valuable features can only be fully harnessed through specific design considerations. Firstly, the bio-inertness of gold has been widely accepted in the past [21,22,23]. Furthermore, gold nanoparticles can also be stimulated via UV and X-ray irradiation to produce ROS which can be carefully tailored for more deliberate biological intentions. This finding therefore elevated gold nanoparticles as a highly useful tool for addressing some of the more challenging biological questions, with cancer therapy remaining high on the list. While the direct administration of gold nanoparticles for PDT-related cancer therapeutic purposes may suffice for some situations, there have been calls for the design of more complex delivery platforms [24,25]. Interestingly, apart from gold-based materials, another important material candidate for cancer nanotherapeutics is mesoporous silica nanoparticles (MSNs) [26,27]. In terms of drug delivery, many groups have sought to utilize mesoporous silica’s high surface area and its bio-inertness to deliver challenging drug molecules, especially those with incompatible solubility [28]. These mesoporous silica-based systems have already been widely reported in literature and have shown much success in targeting many cancer targets so far [28,29,30,31]. Consequently, it is inevitable that both bio-inert materials (gold and mesoporous silica) would ultimately arrive to a synergetic relationship in due course. Intentional hybridization between both systems would confer two excellent attributes to the overall drug design. Firstly, gold nanoparticles can produce a lethal dosage of ROS for cancer targets as well as provide the necessary platform for bioimaging, and mesoporous silica can facilitate a dedicated control release profile for the drug while extending its protection of the drug molecule encapsulated within the core.

**Figure 1:**
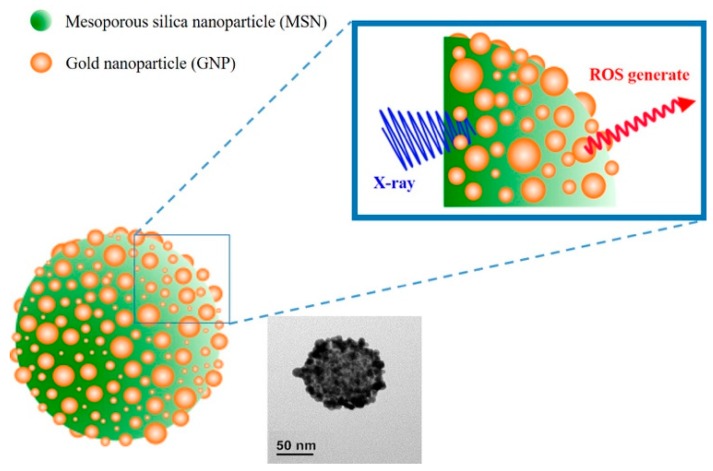
Schematic illustration of X-ray-induced photodynamic therapy (PDT) with pollen-structured gold clusters (PSGCs).

Herein, pollen-structured gold nanoclusters (PSGCs) were designed to mediate X-ray-induced PDT effects (Figure 1). In an attempt to simplify the production of the gold nanoparticle shell-mesoporous silica core hybrid system (refers as PSGCs), herein we report the conceptualization of a simple deposition-precipitation process, based on that of Kah and coworkers, although our system differs in having a pollen-like gold structure on the surface [32]. In brief, the core mesoporous silica was first produced via the conventional Stöber process and terminated with (3-aminopropyl) triethoxysilane (APTES). The surface amines were then passivated with a rich layer of gold nanoparticles via electrostatic interaction and subsequently aged to form a rich pollen-like coverage on the surface of the mesoporous silica core. In order to systematically catalogue the characteristics of these gold nanoparticles, we decided to solely focus on the surface attributes of these gold pollen structures—namely, on the ROS production under the irradiation of a low dosage X-ray. These produced ROS levels were subsequently corroborated with preliminary cell-damage studies to better understand how these systems may work in tandem for subsequent cancer therapeutic discourse.

## 2. Materials and Methods

### 2.1. Materials

The materials employed in this study are listed as follows: tetraethoxysilane (TEOS, 98%, Sigma Aldrich, Darmstad, Germany), ammonium hydroxide (NH_4_OH, Sigma-Aldrich, 30–33%), ammonium nitrate (NH_4_NO_3_, Sigma-Aldrich), 3-aminopropyltriethoxysilane (APTES, Sigma-Aldrich, 99%), chloroauric acid (HAuCl_4_, 99.9%, Alfa Aesar, Haverhill, MA, USA), n-octane (Alfa Aesar), sodium borohydride (NaBH_4_), hexadecyltrimethylammonium bromide (CTAB, Alfa Aesar, 99%), paraformaldehyde (Alfa Aesar, 97%), sodium hydroxide (NaOH), sodium citrate dehydrate (MACRON), potassium carbonate (K_2_CO_3_, J.B. Baker, Phillipsburg, NJ, USA), ethanol (J.B. Baker, 99.5%), dihydroethidium (DHE, Thermo Fisher Scientific, Rockford, IL, USA), wheat germ agglutinate AlexaFluor 488 (WGA-488, Thermo Fisher Scientific), ProLong^®^ Diamond Antifade Mountant (Thermo Fisher Scientific), Hoechst 33342 (AAT bioquest), γ-H2AX antibody, and anti-mouse-AlexaFluor594 (GeneTex, Irvine, CA, USA).

### 2.2. Preparation of Mesoporous Silica Nanoparticles (MSNs)

MSNs were synthesized according to a previous report [27]. Typically, 0.58 g of CTAB was completely dissolved in 300 mL of 0.17 M ammonium hydroxide (NH_4_OH) and 5.0 g of n-octane was added into the mixed solution. After stirring gently at 40 °C for 1 h, 5 mL of 0.2 M tetraethoxysilane (TEOS) was introduced into the mixed solution. Four hours later, 1.0 M TEOS was added dropwise into the mixed solution. The solution was stirred for another 1 h, followed by aging for 24 h at 40 °C. As-synthesized MSNs were then collected and washed by centrifugation (12,000 rpm for 30 min) three times and subsequently dispersed in 99.5% ethanol. For CTAB template removal, the as-synthesized MSNs were refluxed with 250 mg ammonium nitrate in 99.5% ethanol at 60 °C for 24 h.

### 2.3. Synthesis of Pollen-Structured Gold Clusters (PSGCs)

PSGCs were synthesized via a deposition-precipitation (DP) process reported by Kah and coworkers [32]. As a step before PSGCs synthesis, extracted MSNs were functionalized with APTES to yield MSN-NH_2_. To this, 25 mL of MSN in ethanol and 150 μL of APTES were added into a round bottom flask and the reaction was left overnight with vigorous stirring at 70 °C. Next, 0.45 mL of 0.1 M NaOH and 2 mL of 6.35 mM HAuCl_4_ were added simultaneously in a vial. After 15 min of stirring, 1 mL of MSN-NH_2_ (9 mg/mL in deionized water) was added to the suspension and stirred at 70 °C for 30 min. The final products, now referred to as seed particles, were then centrifuged and re-suspended in 4 mL of deionized water. In growing the PSGCs, seed particles were added into the K-gold solution with a volume ratio of seed-to-K-gold of 1:300. To prepare the K-gold solution, 2 mL of 1% (wt) HAuCl_4_ was added to 100 mL of water containing 0.025 g of K_2_CO_3_ under magnetic stirring.

### 2.4. Synthesis of Gold Nanoparticles (AuNPs)

AuNPs (100 nm) were synthesized according to the protocol reported by Ziegler and colleagues [33]. Briefly, AuNPs were prepared by synthesizing seed particles followed by three growing steps. For seed particles, 2.5 mL of 0.2% *w/v* HAuCl_4_ in 50 mL of water was heated up until boiling. After that, 2 mL of 1% *w/v* citrate solution (containing 0.05% *w/v* citric acid) was introduced quickly to the solution and stirring was continued for another 5 min. The forming seed particles were allowed to cool down to room temperature before proceeding to the growing steps. In the first growing step, 3 mL of seed particles in 20 mL of water was added into a three-necked round bottom flask. Solution A (2 mL of 0.2% HAuCl_4_ in 10 mL of water) and solution B (0.5 mL of 1% ascorbic acid and 0.25 mL of 1% sodium citrate in 10 mL of water) were added simultaneously via a peristaltic pump at 0.22 mL/min. Then, the mixed solution (solution I) was heated up to its boiling point and maintained at the boiling point for 30 min. Next, for the second growing step, 4.5 mL of solution I in 20 mL of water was added into a three-necked round bottom flask. Solutions A and B were added simultaneously via a peristaltic pump at 0.22 mL/min. This mixed solution (solution II) was heated up to its boiling point and maintained at the boiling point for 30 min. In the last growing step, 20 mL of solution II was initially placed in a three-necked round bottom flask. Next, solution C (8 mL of 0.2% HAuCl_4_ in 10 mL of water) and solution D (2 mL of 1% ascorbic acid and 1 mL of 1% sodium citrate in 10 mL of water) were added simultaneously via a peristaltic pump at 0.22 mL/min. This mixed solution (solution III) was heated up to its boiling point and maintained at the boiling point for 30 min. The size of the AuNPs in solution III was examined via transmission electron microscope (TEM) and analyzed using ImageJ software.

### 2.5. Quantification of Produced Reactive Oxygen Species (ROS) with Dihydroethidium (DHE)

Dihydroethidium (DHE) was used to quantify the production of superoxide radicals (O_2_^−^) after X-ray irradiation. Prior to X-ray irradiation, 2.4 μL of DHE was added to 400 μL of water, AuNPs (1 mg/mL), and PSGCs (1 mg/mL) in aqueous solution, separately. These mixed samples were then exposed to a variety irradiation doses (0, 0.5, 1, 2, and 5 Gy). X-Ray irradiation was performed in a single dose over an appropriate field size with the setting of 160 keV, 25mA by X-ray irradiator (Rad Source RS2000). Thereafter, the nanoparticles were pelleted and the fluorescence intensity of the supernatant was measured via a fluorescence spectrophotometer (Varian Cary Eclipse) with excitation at 490 nm.

### 2.6. Comet Assay

MD-MBA-231 cells were treated with AuNPs and PSGCs at a concentration of 100 μg/mL in serum-free RPMI-1640 for 2 h prior to X-ray irradiation. Non-treated cells were used as a negative control for this experiment. The cells were exposed to different X-ray irradiation doses (0 and 5 Gy) followed by another 2 h of incubation under normal conditions. After that, the cells were trypsinized and re-suspended in 250 μL of iced phosphate buffered saline (PBS). Then, 50 μL of cells was mixed with 250 μL of LMAgarose at 37 °C. Immediately, 75 μL of the cell-agarose mixture was dropped on a comet assay slide and covered with a coverslip. The prepared slides were kept at 4 °C for 30 min to solidify the mixture. After the allocated time, the coverslips were removed and the slides were immersed in lysis solution for 30 min in 4 °C. After the lysis step, the slides were immersed in alkaline solution for 30 min in the dark. After 30 min, the slides were washed with Tris-borate-EDTA (TBE) buffer (5 min) twice. Then, electrophoresis was performed with TBE buffer for 20 min at 25 V (1.1 V/cm). After that, the slides were dehydrated in 70% ethanol for 5 min and dried in a desiccator. The slides were stained with SYBER GREEN (1:1000) for 30 min in the dark. The ratio of tail DNA to total DNA was quantified using comet assay software (CaspLab) to evaluate DNA damage.

### 2.7. Immunoblotting Analysis

Twenty-four hours prior to the experiment, the cells were seeded at a density of 1.6 × 10^6^ in 6-cm wells. Cells were than incubated with 100 μg/mL of PSGCs for 2 h and exposed to different X-ray irradiation doses (0, 1, 2, and 5 Gy), followed by another 4 h of incubation under normal conditions. The total protein concentration was determined by bicinchoninic acid assay (Bio Rad). Whole cell lysates were resolved by 15% of SDS-PAGE and transferred onto polyvinylidene fluoride membranes (Milipore) using a wet transfer system (Hoefer) with a Tris-glycine buffer containing 20% methanol. The membrane was then blocked with 5% skim milk in Tris-buffered saline with 0.1% Tween-20 (TBST) for 1 h at room temperature and gently washed three to four times with TBST. After that, the membrane was probed with antibodies against Caspase 3 (GTX110543) and β-actin antibody (GTX109639) for 16 to 18 h at 4 °C and washed three times for 5 min with TBST. Finally, the blot was detected with HRP-linked Anti-rabbit IgG antibodies (GTX213110-01) for 50 min at room temperature, followed by three washes with TBST for 5 min. The blot was developed using Immobilon Western Chemiluminescent HRP Substrate (Milipore) and quantitatively analyzed using ImageJ software.

### 2.8. Immunofluorescence Staining

The X-ray irradiation treatments were similar to the Comet assay described above. After irradiation, cells were washed three times with PBS and fixed with 4% paraformaldehyde. After fixation, the cells were washed three times with PBST. Next, 5% of bovine serum albumin (BSA) in PBST was used for blocking for 1 h. After blocking, the cells were incubated with primary antibody, γ-H2AX (1:2000), at room temperature for 1 h. The primary antibodies were detected by fluorescent secondary antibody, anti-mouse-AlexaFluor594 (1:500). Then, cells were counterstained with wheat germ agglutinin-AlexaFluor 488 (WGA-488) and DAPI for 5 min each. Afterwards, the cells were washed with PBST and the coverslips (cell side-down) were sandwiched on a microscope slide loaded with a drop of ProLong^®^ Diamond Antifade Mountant (Thermo Fisher Scientific, Rockford, IL, USA). The samples were then ready to be viewed under a confocal microscope (Leica TCS SP2).

## 3. Results and Discussions

**Figure 2:**
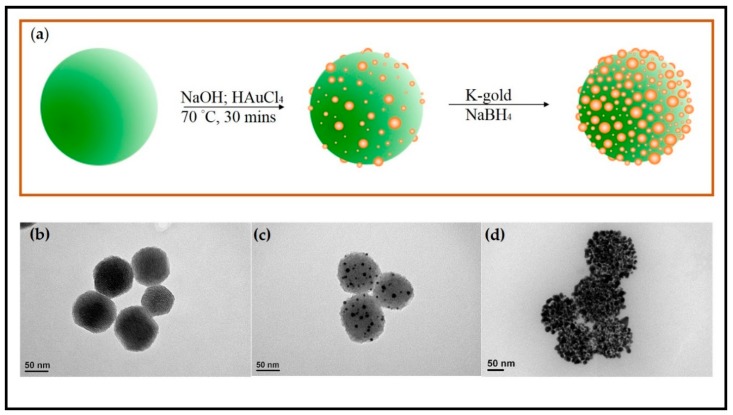
(**a**) Schematic illustration the synthetic route of pollen-structured gold clusters (PSGCs). TEM images of (**b**) mesoporous silica nanoparticle (MSN)-NH_2_, (**c**) seed particles, and (**d**) PSGCs.

The PSGCs, used as PS for X-ray-induced PDT, were synthesized based on the procedure schematically shown in Figure 2a. In brief, MSNs were prepared via the Stöber process as reported previously and CTAB templates were subsequently removed by refluxing with ammonium nitrate [30]. MSNs were selected as the template for gold deposition due to several advantages, including their ease of functionalization, controllable size, and good biocompatibility. Prior to the incorporation of gold on the MSNs surface, functionalization with APTES was performed to obtain a NH_2_-rich layer, which served as the initial site for the growth of PSGCs. Afterward, a one-pot synthesis method to prepare small gold particles with a size of approximately 7.56 ± 2.56 nm as well as the deposition of small gold particles on MSN-NH_2_ were conducted under basic conditions. The gold-deposited MSNs, referred as seed particles, were then mixed with K-gold solution and reduced by NaBH_4_, forming PSGCs. The morphology and size of MSN-NH_2_ (Figure 2b), seed particles (Figure 2c), and PSGCs (Figure 2d) were characterized by TEM. The average particle size of the MSNs was 77.24 ± 9.02 nm, while PSGCs were calculated to be 101 ± 16.3 nm. As shown in Figure 2d, the MSNs surface was evenly covered by gold particles, which formed pollen-like clusters at the end of the synthesis process.

The effectiveness of PDT was dependent on the production of ROS, which subsequently cause oxidative stress beyond the reduction capacity in cancer cells, ultimately resulting in cell death. Thus, the ROS generation efficacy of synthesized PSGCs was assessed comparatively with the same size of spherical AuNPs, approximately 100 nm. To quantify the ROS level produced under X-ray irradiation, DHE, a fluorescent probe for O_2_^−^, was employed to detect and measure the amount of O_2_^−^ in solution under various X-ray dosages (0.5, 1.0, 2.0, and 5.0 Gy). In the presence of O_2_^−^, DHE was oxidized and yielded two products, 2-hydroxyethidium (2-OH-E^+^) and ethidium (E^+^), which emit red fluorescence [34,35]. The increase of the red fluorescence when DHE was oxidized by the generated O_2_^−^ during X-ray irradiation was usually used to quantify the amount of yielded O_2_^−^.

**Figure 3:**
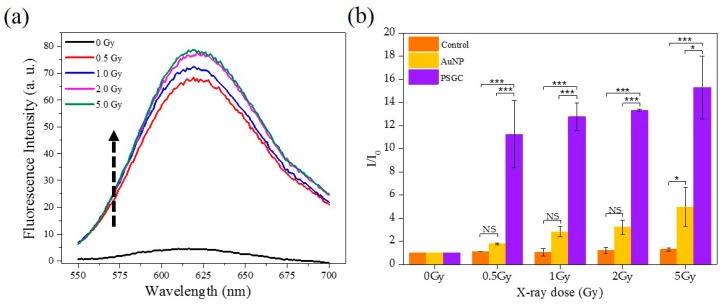
(**a**) Fluorescence emission spectra of dihydroethidium (DHE) in PSGCs aqueous solution with different irradiation doses. (**b**) DHE fluorescence intensity enhancement for water, gold nanoparticles (AuNPs), and PSGCs with a variety X-ray irradiation doses. Statistical significance is denoted as follows: * represents a *p* value ≤ 0.05 and *** represents a *p* value ≤ 0.001. NS indicates that a value is not statistically significant.

**Figure 4:**
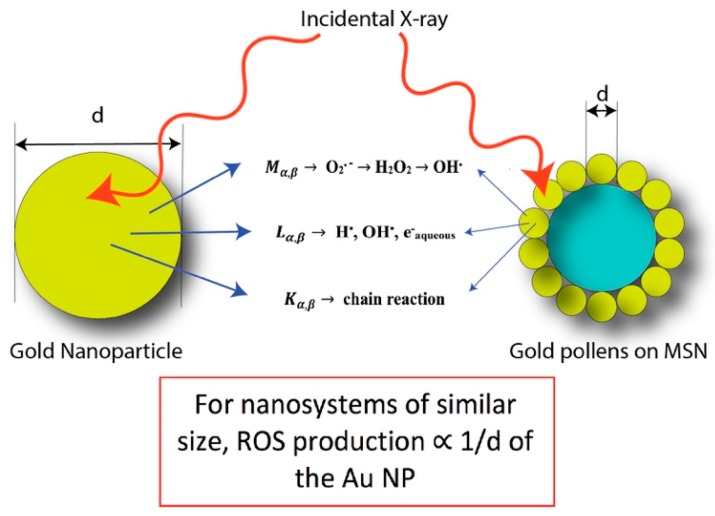
Graphical description of the production of reactive oxygen species (ROS) as a function of surface area and Au diameter, d.

Figure 3a shows an increasing course of DHE fluorescence with the various X-ray irradiation dosages for PSGCs. From the fluorescence emission spectra, there was a significant increase of the fluorescence intensity with respect to the intensity of the irradiation, although a direct proportional relationship was not observed between the dosage and the ROS produced. Rather, the maximum fluorescence was noticed to have peak between 2 and 5 Gy, and this strongly suggested that the full potential of ROS production for these pollen-like structure could have been attained at dosages as low as 2 Gy. The comparison of DHE increment by water, AuNPs, and PSGCs were subsequently quantified and are shown in Figure 3b. DHE fluorescence intensity elevated significantly in AuNPs and PSGCs as the radiation dose increased. A nearly 3-fold increase of O_2_^-^ generation was observed in PSGCs compared to AuNPs when exposed to a 5 Gy dose of X-ray. With the same particle size of AuNPs and PSGCs, PSGCs have a higher surface area to catalyze the ROS generation upon X-ray irradiation and PSGCs were found to be statistically significant across the spectrum when compared to AuNPs. This observation was consistent with the notion that ROS is directly proportional to the 1/d, where d represents the diameter, as described in Figure 4, which also closely mirrors the report by Misawa et al. [36]. Although it is necessary to note that the DHE production at 2 Gy and 5 Gy was not found to have much statistical significance, this is also in agreement with our previous observation on ROS production.

Oxidative stress created during X-ray-induced PDT has been recognized as the primary cause of DNA double strand breaks (DSBs). In response to DSBs, histone H2AX is phosphorylated to γ-H2AX, which plays a crucial role in enrolling and localizing the DNA repair mechanism [37,38]. Therefore, this protein is deemed suitable as a biomarker for detecting DSBs. Herein, immunofluorescence staining with γ-H2AX was performed at the cellular level to further evaluate the DNA damage after X-ray irradiation with either the presence or absence of PSGCs. Furthermore, we also monitored the downstream apoptosis protein expression to assess the PDT effect. Caspase 3 is known to play a key role in the execution of apoptosis in vertebrate cells. The immunoblotting analysis of activated caspase 3 was demonstrated to further confirm the intracellular ROS formation and ROS-mediated cell death.

**Figure 5:**
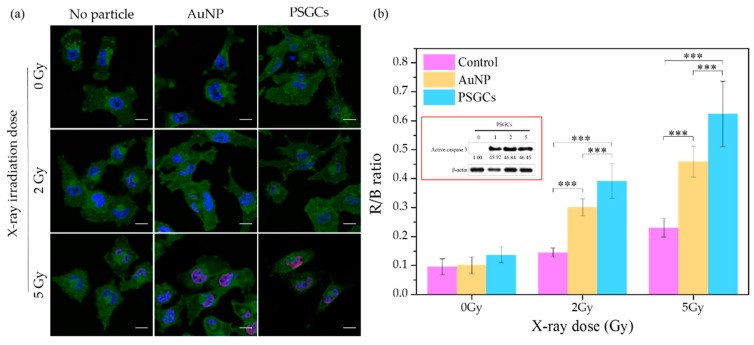
(**a**) Immunofluorescence staining of γ-H2AX (red) for MD-MBA-231 cells treated with AuNPs and PSGCs after 0, 2, and 5 Gy X-ray irradiation. Scale bar is 15 µm. (**b**) Quantitative fluorescence intensity ratio of γ-H2AX to nucleus (red/blue, R/B) for MD-MBA-231 cells treated without and treated with AuNPs and PSGCs after irradiation at 0, 2, and 5 Gy. Inset shows the immunoblotting analysis of caspase 3 protein expression for cells treated with PSGCs after 0, 1, 2, and 5 Gy X-ray irradiation. Statistical significance is denoted as follows: *** represents a *p* value ≤ 0.001. NS indicates that a value is not statistically significant.

Figure 5a illustrates confocal images of MD-MBA-231 cells stained with γ-H2AX, which localized in the nucleus after a single irradiation dose at 2 Gy and 5 Gy, correspondingly. The distribution of γ-H2AX (red) throughout the nucleus (blue) was quantified by analyzing the fluorescence intensity of the red to blue (R/B) ratio with ImageJ software, and it was then plotted against X-ray dosage (Gy) as shown in Figure 5b. As compared to untreated cells and AuNP-treated cells, the strongest signal of γ-H2AX was detected in PSGC-treated cells with irradiation. In addition, caspase 3 protein expression for PSGC-treated cells with irradiation was also demonstrated. β-actin protein expression was used as the loading control to normalize the possible errors from the whole process of immunoblotting. The inset in 5b shows a high caspase 3 protein expression level at 4 h after X-ray irradiation at 1, 2, and 5 Gy. The results imply that cells were damaged and had undergone the apoptosis process at this time point, even with a low dosage of X-ray irradiation. Taken together, the in vitro cell study findings were highly correlated with the results from the DHE assay, further confirming the potential of PSGCs in X-ray-induced PDT. Although we noticed that while ROS production was found to have no statistical significance between 2 Gy and 5 Gy, the cellular data seems to suggest that 5 Gy was more efficient in terms of γ-H2AX analysis. This result should be taken into consideration, suggesting that X-ray irradiation alone may induce minor cellular DNA damage. Thus, at close scrutiny of the untreated control samples, we noticed that there was only a slight augmentation of the R/B ratio with respect to the X-ray dosage increment. While the increase of γ-H2AX levels in this study were not found to be statistically significant in all untreated controls for the various X-ray dosages, the marginal increment should be factored for PSGCs, contributing slightly to the higher levels as observed for the PSGCs. Nonetheless, the lack of an appreciable ROS level for the untreated control with respect to the X-ray dosage is highly encouraging and this strongly reinforces the notion of that the novel PSGC system described here may have high clinical practicality.

**Figure 6:**
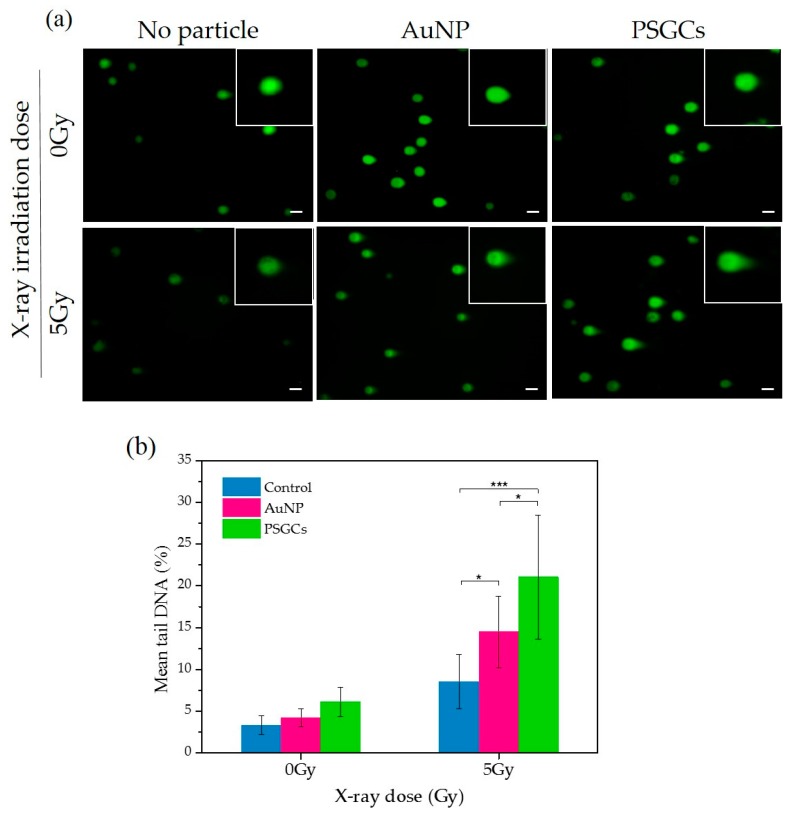
(**a**) Evaluation of effectiveness X-ray-induced PDT via comet assay. Scale bar is 10 µm. (**b**) The DNA damage was detected by quantifying the mean tail DNA (%) and was plotted against X-ray doses. Statistical significance is denoted as follows: * represents a *p* value ≤ 0.05 and *** represents a *p* value ≤ 0.001. NS denotes that a value is not statistically significant.

Comet assay, also known as single cell gel electrophoresis (SCGE), was carried out with MDA-MB-231 cells to further confirm the DNA damage via X-ray-induced PDT with the presence of AuNPs and PSGCs, separately. The degree of DNA damage was quantified by evaluating the percentage DNA in tail with comet assay software, and quantitative data were then plotted against X-ray dosage (Gy), as shown in Figure 6b. In Figure 6, PSGC-treated cells under 5 Gy of X-ray displayed 20.12 ± 9% DNA in tail, which is two times higher than that achieved with X-ray treatment alone. Furthermore, DNA tail was also observed with AuNP-treated cells plus 5 Gy X-ray, but the percentage of DNA tail was in between that achieved with X-ray alone and with PSGCs and X-ray, at a value of nearly 14.6 ± 4.21%. The efficacy of X-ray-induced PDT was found to be directly proportional to the 1/d, as described above in Figure 4. With same size of nanosystem, PSGCs can generate more ROS than AuNPs because of their greatly enhanced surface area. In vitro DHE assay and cellular level DNA damage results including γ-H2AX staining and comet assay all correlated with each other and supported this hypothesis.

## 4. Conclusions

The design of PSGCs was intended to demonstrate the feasibility of using MSNs as a carrier for PS-serving gold nanostructures, and this work represents an important advancement in this aspect. Localizing gold particles on a single carrier would sometimes be more advantageous than the administration of free-moving gold particles in a physiological environment due to the potential haphazardness of non-localized nanoparticles that could cause collateral damage to healthy tissues. In addition, with the same size of nanosystem, PSGCs have a greater surface area than spherical AuNPs, which is a critical factor for the efficacy of X-ray-induced PDT. We demonstrated that gold particles organized in pollen-like nanostructures were more efficient in generating ROS, and this could imply the potential clinical translation of PDT for deep-tissue cancer treatment. When applying X-ray for PDT, caution must be taken as X-ray alone could have indiscriminate damaging effects. However, our PSGCs nanostructure was found to be as effective between 2 and 5 Gy, which is a fraction of the radiation dosage administered during conventional cancer radiotherapy; the results herein were highly promising in this aspect. As a proof of concept, the PSGCs were shown to be an excellent mediator for ROS at an X-ray radiation dose way below the nominal threshold of causing extensive cellular damage. Thus, future work will encompass the applicability of such nanostructures in the standard in vivo environment.
